# Critical care considerations in the management of the trauma patient following initial resuscitation

**DOI:** 10.1186/1757-7241-20-68

**Published:** 2012-09-18

**Authors:** Roger F Shere-Wolfe, Samuel M Galvagno, Thomas E Grissom

**Affiliations:** 1 University of Maryland School of Medicine, R Adams Cowley Shock Trauma Center, 22 S. Greene St, Ste. T1R77, Baltimore, MD, 21201, USA

**Keywords:** Coagulopathy, Trauma, Acute lung injury, Transfusion, Intensive care unit, Complications, Thromboelastography

## Abstract

**Background:**

Care of the polytrauma patient does not end in the operating room or resuscitation bay. The patient presenting to the intensive care unit following initial resuscitation and damage control surgery may be far from stable with ongoing hemorrhage, resuscitation needs, and injuries still requiring definitive repair. The intensive care physician must understand the respiratory, cardiovascular, metabolic, and immunologic consequences of trauma resuscitation and massive transfusion in order to evaluate and adjust the ongoing resuscitative needs of the patient and address potential complications. In this review, we address ongoing resuscitation in the intensive care unit along with potential complications in the trauma patient after initial resuscitation. Complications such as abdominal compartment syndrome, transfusion related patterns of acute lung injury and metabolic consequences subsequent to post-trauma resuscitation are presented.

**Methods:**

A non-systematic literature search was conducted using PubMed and the Cochrane Database of Systematic Reviews up to May 2012.

**Results and conclusion:**

Polytrauma patients with severe shock from hemorrhage and massive tissue injury present major challenges for management and resuscitation in the intensive care setting. Many of the current recommendations for “damage control resuscitation” including the use of fixed ratios in the treatment of trauma induced coagulopathy remain controversial. A lack of large, randomized, controlled trials leaves most recommendations at the level of consensus, expert opinion. Ongoing trials and improvements in monitoring and resuscitation technologies will further influence how we manage these complex and challenging patients.

## Introduction

Resuscitation of the severely injured patient is a topic of ongoing evolution and controversy. Since the early 1990’s, management of critically ill polytrauma patients has been governed by the “damage control” paradigm first introduced in abdominal surgery [1] and subsequently expanded to most areas of care, including orthopedic [2], vascular [3] and thoracic injuries [4]. According to one definition, damage control surgery (DCS) is the “planned temporary sacrifice of normal anatomy to preserve vital physiology”[5]. Because severely injured patients are too physiologically deranged to tolerate prolonged definitive repair, initial surgical intervention is limited to minimally necessary stabilization and control of hemorrhage. Thus, the patient presenting to the intensive care unit (ICU) following initial resuscitation and DCS may be far from stable with ongoing hemorrhage, resuscitation needs, and injuries still requiring definitive repair.

Care of the polytrauma patient does not end in the operating room or resuscitation bay. As one authority has noted, “the best place for a sick person is in the ICU”[6]. ICU physicians must be prepared to receive patients at any point along the continuum of care, and must be adept at assessing the patient’s physiologic status and addressing ongoing needs in a prompt and expeditious fashion. As the patient stabilizes, the ICU physician must then begin to transition the focus of care to longer term considerations such as potential for infectious and thromboembolic complications, organ support, and the need for planned re-exploration and staged definitive repair.

Although much focus has been placed on the initial management of the traumatized patient, the transition between early resuscitation of the critically injured patient with hemorrhage and/or polytrauma and the ICU has received less attention. These patients pose a number of unique challenges for the ICU physician including the need for ongoing resuscitation, determination of resuscitation endpoints, and management of early post-resuscitation complications. How well these are addressed may have critical implications for long-term outcome and survival. In this review, we will address early ICU considerations in the polytrauma patient requiring aggressive early resuscitation. While no consensus definition for “polytrauma” has been recognized, generally accepted definitions use an Injury Severity Score (ISS) of greater than 15 to 17 or an Abbreviated Injury Scale (AIS) of greater than 2 in at least two body regions [7].

### Continued resuscitation in the ICU

#### Immediate assessment and basic physiologic support

**Figure 1:**
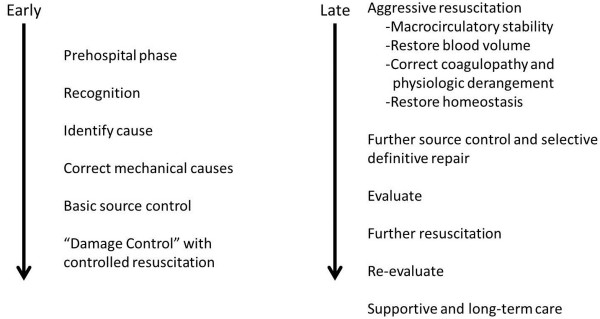
A general approach to early versus late resuscitation.

**Figure 2:**
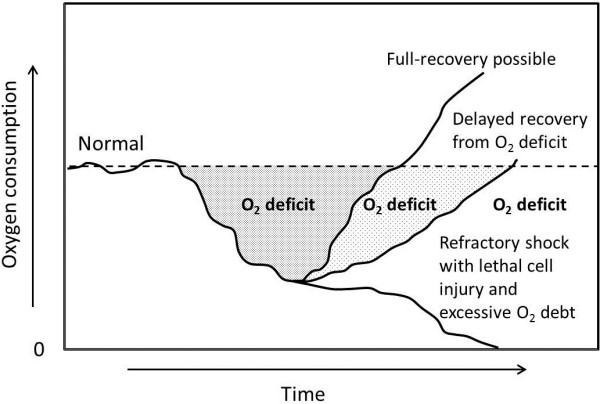
**Prolonged tissue hypoperfusion creates a cumulative “oxygen debt” directly related to the “dose” of shock, based on both the duration and depth of hypoperfusion.** Eventually this results in irreversible disruption of homeostasis such that patients will not respond to resuscitative efforts even after the initial insults have been corrected [adopted from [9]].

Upon arrival to the ICU it is essential for the ICU physician to understand where the patient is in the continuum of both surgical management and ongoing resuscitation (Figure 1), and to assess overall stability and the extent of unresolved shock. Because shock is a cumulative phenomenon in which the depth and duration determine the total “dose” in an integrative fashion [8], the timeliness of resuscitation may have a significant impact on subsequent morbidity and mortality (Figure 2) [9]. Virtually all critically injured patients require some degree of immediate physiologic support on arrival to the ICU. This includes assurance of adequate respiratory and ventilator support as well as aggressive intervention to minimize secondary central nervous system (CNS) injury, resolve critical acid–base and electrolyte disorders and restore normothermia.

Volume loading remains the mainstay of circulatory support. Vasopressors seldom improve microvascular perfusion and may mask underlying shock so their early role in the resuscitation of the trauma should generally be cautioned [10]. Patients requiring vasopressors are often either severely physiologically perturbed or under-resuscitated, and their lack of response to fluid therapy may be suggestive of irreversible shock. There has been some recent support for the use of low-dose vasopressin to treat underlying deficiency and decrease overall fluid requirements [11-13] though this is not universally accepted, and may impair the micro-circulation and produce splanchnic ischemia [14]. Patients with concomitant CNS injuries may require vasopressor support to counteract spinal shock, or to maintain cerebral perfusion in the setting of traumatic brain injury (TBI).

Respiratory support must continue to ensure adequate oxygenation and ventilation. Inadequate oxygen delivery only worsens tissue hypoperfusion. This may be especially deleterious in cases with concomitant CNS injury [15]. Respiratory acidosis superimposed on metabolic acidosis may also be extremely detrimental. The use of positive end-expiratory pressure and open lung ventilation techniques in the hypovolemic patient can increase intrathoracic pressure and may critically impede venous return resulting in profound hypotension. Physiologically deranged patients often present to the ICU with profound metabolic acidosis and hypothermia. These impair both hemodynamic and hemostatic function. Hypothermia should be corrected aggressively with full body passive or active re-warming. Metabolic acidosis predicts both mortality and transfusion needs [16,17], and is generally best treated by restoration of tissue perfusion. Massive fluid shifts often produce profound electrolyte disturbances, which should also be promptly corrected.

Polytrauma patients with concomitant CNS injuries pose an especially great challenge. Even mild TBI can blossom into a life-threatening condition when compounded by hypoxia and hypotension [15]. Prevention of secondary injury should be among the highest priories in any patient with evidence or suspicion of CNS injury [18]. CNS assessment and/or monitoring should be instituted at the earliest possible juncture.

#### Assessment of hemostasis and correction of coagulopathy

Cessation of bleeding, whether surgical or medical, is the *sine qua non* of resuscitation from injury. There is little utility in targeting endpoints of resuscitation in the face of ongoing hemorrhage. Life-threatening coagulopathy is one of the most serious complications of patients in profound shock from massive hemorrhage, and is generally predictable at an early stage [19]. Increased early transfusion requirements are also generally predictive of subsequent organ dysfunction [20-22]. Studies have shown that ongoing coagulopathy on admission to the ICU is independently associated with both an increase in morbidity and 30-day mortality [23].

The majority of trauma patients initially present with normal or prothrombotic coagulation profiles. However, those most seriously injured are likely to present with evidence of hypocoagulability, accelerated fibrinolysis, or both [24,25]. Upon transfer to the ICU the patient’s coagulation status may be in any of these states. It is essential therefore to promptly re-assess the patient’s coagulation status in order to initiate appropriate therapy. “Standard” laboratory tests such as prothrombin time (PT), partial thromboplastin time (PTT), international normalized ratio (INR), fibrinogen level and platelet count are still the most common coagulation assays in clinical use, despite considerable evidence that they provide an extremely incomplete picture of *in vivo* hemostasis [26,27], that they are poor predictors of clinical bleeding [28], and that they do not provide an adequate basis for rational targeted hemostatic resuscitation [29,30]. Although significantly elevated admission PT and PTT levels are predictive of increased mortality from injury [31], there is little evidence that they provide a realistic target for resuscitation. Moderately elevated values may have little clinical significance, and correction to “normal” values may require large amounts of resuscitation fluids, especially fresh frozen plasma (FFP). In the absence of active clinical bleeding, attempts to normalize laboratory values have the potential to introduce transfusion- and volume-related complications

These deficiencies underscore the need for reliable point-of-care hemostatic monitoring with clinical relevance in situations of generalized coagulopathy due to massive hemorrhage. There is increasing evidence that viscoelastic monitoring technologies such as TEG^®^ (Haemonetics Corp., Niles, IL, USA) and ROTEM^®^ (Tem Innovations GmbH, Munich, Germany) are superior for detecting clinically relevant hemostatic abnormalities in trauma and surgical patients with massive bleeding and diffuse coagulopathy [32,33]. Viscoelastic monitoring has been much more widely used in Europe than in the United States, for both intra-operative and ICU management of bleeding surgical and trauma patients. Schöchl and colleagues have recently published a detailed review on the use of viscoelastic monitoring targeted resuscitations [34]. It should also be noted that both viscoelastic and standard coagulation tests are generally performed after warming specimens to 37°C, and do not reflect the potentially considerable effects of hypothermia on *in vivo* hemostasis [35].

**Figure 3:**
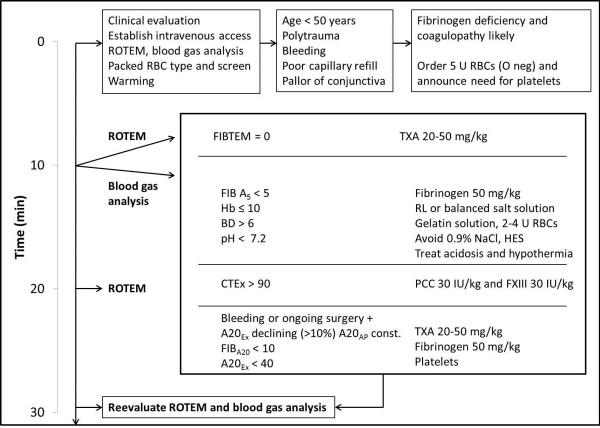
**One possible decision tree algorithm for the management of clinical bleeding using ROTEM^®^-guided goal-directed resuscitation with targeted hemostatic factors [adopted from **[27].

Because of evidence that severely injured trauma patients are likely to develop an early and aggressive endogenous coagulopathy separate from later loss and dilution of clotting factors compounded from hypothermia and acidosis [31,36-41], the practice of “hemostatic” resuscitation has become commonplace in the most severely injured patients. This entails the early and aggressive use of hemostatic products combined with red blood cells as the primary resuscitation fluids in order to avoid rapid deterioration into the “bloody vicious cycle” and the classic “lethal triad” of hypothermia, acidosis and coagulopathy [42]. Two very distinct paradigms of hemostatic resuscitation have currently emerged: the damage control resuscitation (DCR) model, which uses pre-emptive administration of empiric ratios of blood and hemostatic products to approximate whole blood, often according to an established institutional “massive transfusion protocol” [43-47]; and goal-directed hemostatic resuscitation approaches (also often protocol-based), which generally use point-of-care viscoelastic monitoring (Figure 3) combined with the prompt administration of hemostatic concentrates [24,26,27,34]. Regardless, it is highly likely that the patient with massive hemorrhage who arrives to the ICU under-resuscitated with a coagulopathy has been managed according to some sort of hemostatic resuscitation approach which should be continued in the ICU until it is clear that hemostasis has been achieved. It is beyond the scope of this review to discuss the relative merits of these two approaches in detail, however, the critical care provider should communicate with the trauma and operative team to see where the patient is in terms of their hemostatic resuscitation.

With DCR, large volumes of fresh frozen plasma are frequently administered as part of the hemostatic resuscitation and this aggressive use of FFP may be continued into the ICU setting. This aggressive use of FFP may substantially increase the risks of adverse complications. One study attempting to correct the INR to 1.3 in the ICU documented a high rate of severe ARDS [48,49]. Isolated PT/INR levels are poor predictors of clinical bleeding in trauma patients, and thrombin generation is generally preserved or even increased after significant blood loss because of dysregulation, with loss of clotting factors balanced by loss of regulatory inhibitors [50]. If FFP is being used as the primary hemostatic resuscitation fluid and viscoelastic monitoring is not available, the ICU physician should generally accept an INR in the 1.5-1.7 range provided there is no evidence of active bleeding. This INR target is based on studies demonstrating a limited ability of FFP transfusions to normalize coagulation test results with an INR < 1.7 [51].

Recombinant activated factor VIIa (rFVIIa) has found considerable off-label use in the management of refractory coagulopathy in hemorrhagic shock/trauma patients. Although initially touted as a “total hemostatic agent”, it now seems clear that rFVIIa acts mainly as a potent thrombin generator [52]. High dose rFVIIa (usually > = 80–90 μg/kg) works predominantly by a direct effect on activated platelets rather than via its higher affinity binding to tissue factor [53]. Because tissue factor is expressed by inflammatory cells, there is significant potential for systemic micro-thrombi generation, and several studies have shown a risk of thrombotic complications on the order of 5–7% [54]. Efficacy of rFVIIa is dependent on the presence of adequate substrate for clot formation such as fibrinogen and platelets, and may be significantly impaired under acidotic conditions [55]. Because of these considerations, the use of high dose rFVIIa as rescue therapy in refractory hemorrhagic shock is controversial, and should be undertaken with caution. The ICU physician should be aware that patients who have received rFVIIa during their resuscitation may have a normal INR upon arrival to the ICU, but this may only be a transient finding.

In addition to the coagulopathy associated with major trauma, fibrinolysis is especially deleterious in severely injured trauma patients and carries an associated mortality well upwards of 50% [24,56,57]. Many patients with primary fibrinolysis from severe hemorrhagic shock may never survive to reach the ICU. The recently concluded CRASH-2 trial is the only class I evidence to date showing a 30 day survival benefit for a resuscitative therapy [58]. Subgroup analysis showed that the benefit was greatest when therapy was instituted within 1 hour of admission. However, subgroup analysis showed that mortality actually increased when therapy was instituted after 3 hours, suggesting that the risks of therapy outweighed the benefits in patients who survived beyond that timeframe [59]. It may therefore be prudent to carefully consider whether to administer anti-fibrinolytic therapy in the ICU, even if the patient has laboratory evidence of fibrinolysis.

Finally, trauma patients frequently convert from a hypocoagulable to a hypercoagulable profile once they survive the initial insult and hemorrhage [60]. This is important to monitor in the ICU, as long term morbidity and mortality from thromboembolic events has a significant impact; and it is important to discontinue hemostatic support once the patient is no longer coagulopathic.

#### Fluid support in the ICU

**Figure 4:**
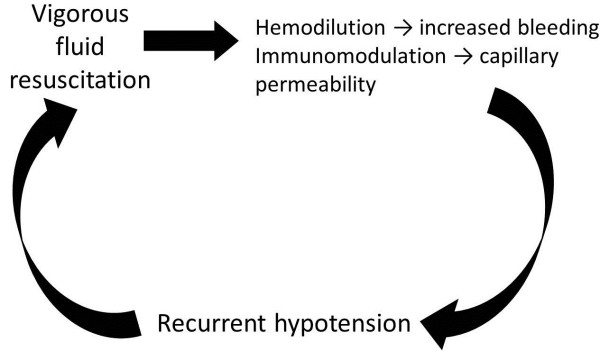
Potential impact of overaggressive fluid administration.

Until definitive hemostasis has been achieved in the ICU, use of non-hemostatic/non-oxygenating fluids should generally be minimized, unless concentrates are used. Resuscitation fluids should consist mainly of blood products and hemostatic agents. Over-aggressive fluid administration in the bleeding patient can lead to clot disruption and exacerbate hemorrhage, resulting in the vicious cycle of increased blood loss and fluid administration known as “fluid creep” (Figure 4). Moderate hypotension during the period of “early resuscitation” is generally acceptable in our experience, with possible adjustment for patients with pre-existing cardiac dysfunction or co-existing CNS injury [8,29]. Transfusion should aim for a hemoglobin between 8 and 10 g/dL while the patient is actively bleeding, to provide a margin for error and also to support hemostasis [61].

Once definitive hemostasis has been achieved, the patient may still be significantly hypo-perfused. Prolonged activation of the noradrenergic axis results in profound vasoconstriction, which may be aggravated by hypothermia. Hypoperfusion impairs cellular energetics and results in loss of endothelial integrity. Inflammatory cytokines and ischemia-reperfusion injury may result in cellular edema reducing the lumen of capillaries and producing the “no-reflow” phenomenon [8]. Full resuscitation of the severely injured patient requires not only arrest of bleeding and restoring hemodynamic stability but also re-establishing micro-circulatory flow, restoring end-organ homeostasis, and repaying the “oxygen debt”. Failure to do this may result in the development of subsequent organ dysfunction in the hemodynamically stable but still under-resuscitated patient [62]. Therefore, assuring adequate completeness of resuscitation is the next critical challenge for the ICU physician after establishing hemostasis. Unfortunately, over-resuscitation as well as under-resuscitation may have adverse consequences, and the “optimal” endpoint of resuscitation may not be at all obvious.

Fluid selection may have implications for micro-circulatory perfusion, which should be the primary goal of resuscitation once hemostasis has been attained. Some data suggest that fluids exert micro-circulatory effects independent of volume expansion or oxygen-carrying capacity [63-65]. There is some evidence that hyperviscous solutions such as hydroxyethyl starch 130/0.4 or hypertonic solutions such as 7% saline with dextran may have a greater benefit in re-establishing microvascular perfusion than standard crystalloids [65-68], and that the micro-circulatory benefit may be limited to a relatively small initial bolus [65]. “Small volume resuscitation” has been studied more in the pre-hospital and early stages of resuscitation as an adjunct to hypotensive resuscitation [8,14,29,69], further study is required to understand the effects of different resuscitation fluids on microvascular perfusion separate from their effects as volume expanders and their potential role in the ICU resuscitation phase. A detailed review of fluid selection for the post-resuscitation trauma patient is beyond the scope of this review and available elsewhere [70].

#### Need for further interventions

In some cases it may be extremely difficult to differentiate surgical from coagulopathy-associated bleeding. Continuing transfusion requirement and evidence of ongoing blood loss in the setting of aggressive corrective efforts usually imply ongoing surgical bleeding, irreversible shock, or profound hepatic dysfunction. Because blood products generally increase the risk of infection, organ failure, and mortality, and because of the cumulative effect of ongoing shock, it may be prudent to set a limit on ongoing transfusion requirements before mandating surgical or angiographic re-evaluation of the patient to rule out occult injury. The ICU physician should discuss this issue at an early juncture with the surgical team. Wounds with particularly difficult anatomy and a propensity for missed injuries should prompt even greater vigilance.

### Monitoring, assessment, and endpoints of resuscitation in the ICU

#### Endpoints of resuscitation

While the patient is actively bleeding – whether from surgical or medical causes – resuscitation is aimed at minimally acceptable levels of organ perfusion and homeostasis, with the goal of avoiding irreversible shock while not exacerbating hemorrhage. It is not possible to focus on definitive “endpoints” when the target is still moving. Physiologic assessment and resuscitative efforts are focused during this stage on hemostasis and on attaining basic goals with respect to temperature, acidosis, urine output and hemodynamics [8].

Once hemostasis has been attained, resuscitation should aim at the complete restoration of macro- and micro-circulatory stability and of end-organ homeostasis in the ICU. Although not directly supported by randomized trials, this may be achieved with additional fluid administration to restore circulating blood volume, in combination with analgesic and sedative agents to dilate constricted blood vessels and improve microvascular perfusion. Basic hemodynamic goals include a stable systolic pressure > 100 mm Hg and a heart rate less than 100 bpm. Urine output should be normal. Normalization of pH, lactate and base deficit are all suggestive of restored micro-circulatory perfusion [8,29]. Aggressive correction of any residual hypothermia and coagulopathy should also be priorities during this phase. These goals may need to be modified based on patient co-morbidities and/or the presence of concomitant CNS injury.

How much fluid loading is beneficial is a matter of debate. Numerous studies have shown that under-resuscitation results in “occult” or “cryptic” shock, a state of compensated shock, which predisposes to organ dysfunction in the ICU [62,71]. Some series have found that 85% of severely injured patients with normal hemodynamics may be hypoperfused [72,73]. Studies have consistently indicated that persistent elevations of serum base deficit or lactate levels are suggestive of occult hypoperfusion and are predictive or poor outcome in critically ill patients [16,62,74-76]. Some authors have proposed that lactate is a better marker of occult hypoperfusion than base deficit in this population [77]. A recent study found that using serial serum lactate levels to guide treatment of critically ill patients reduced overall in-hospital mortality [78].

The above data, combined with observations that survivors of critical injury tended to exhibit hyperdynamic (or “supranormal”) cardiac output [79] and that young severely injured trauma patients frequently manifested significant occult myocardial dysfunction [72]. led to the practice of aggressive volume loading augmented by the use of inotropes to achieve pre-specified goals for oxygen delivery and cardiac function in the ICU [80-86]. These goals were often difficult to achieve, required substantial volume loading and pharmacologic support, and had mixed results [86-90] with a high rate of intra-abdominal hypertension, pulmonary dysfunction, and other complications [69,91]. Although there seems to be some evidence that patients who are able to mount a hyperdynamic response to injury may have better outcomes [92], current evidence suggests that supportive care with fluids to “normal” resuscitation endpoints produces equivalent results to goal-directed resuscitation to preset “supranormal” DO_2_ values, and avoids the adverse consequences of over-resuscitation [90]. Taken together, it appears that the physiologic cost of supranormal resuscitation is high, and the results at the micro-circulatory level are too questionable to support routine use of this approach.

The particular endpoint chosen may also play a role [92-95]. Mixed venous oxygen saturation [84]. central venous oxygen saturation [96] and left ventricular function [97-99] have all been studied as possible endpoints, as have indicators of regional perfusion. In all likelihood, the majority of the time these are functionally equivalent: responders tend to do well by all endpoints, and non-responders tend to do poorly [14]. It is unclear how useful these are as specific targets for resuscitation, as opposed to simply being markers of physiologic response.

**Figure 5:**
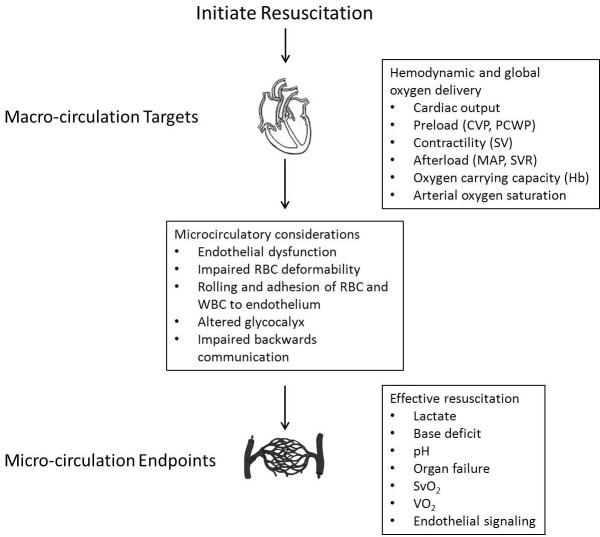
**Macro- and micro-circulatory endpoints for resuscitation [adopted from **[95,100].

Supranormal and goal-directed approaches to resuscitation presume that attaining macro-circulatory targets such as cardiac output and oxygen delivery will directly lead to perfusion at the level of the micro-circulation (Figure 5) [100]. It is far from clear that this is actually the case. Evolving evidence suggests that beyond a minimal level of cardiac output and arterial pressure, there may be considerable disassociation between the micro- and macro-circulation [101,102]. Several studies have shown not only a lack of coupling between hemodynamics and the microcirculation, but also considerable individual variation in the microvascular response to interventions targeting upstream endpoints [103-105] .

In summary, the patient in whom hemostasis is achieved early, limiting the dose of shock and the extent of underlying organ dysfunction, may respond well to aggressive fluid loading to restore tissue perfusion; whereas the patient with prolonged hemorrhage and shock resulting in significant organ dysfunction may not. While a specific, targeted endpoint for resuscitation is important for guiding subsequent therapy, the actual endpoint selected may not be important as the use of goal-directed approach. Other considerations may influence the ICU physician’s approach in specific cases. Regardless, it is evident that there is considerable inter- and intra-patient variation in the Frank-Starling curves of fluid responsiveness [69], and therapy must be carefully tailored to individual needs and responses.

#### Missed injuries and determinants of futility

Not all patients respond to aggressive resuscitative measures. This can be due to occult injury or poor physiologic response. A recent review of undiagnosed injuries and outcomes, suggested up to 6.5% of all trauma-related deaths were attributable to clinically undiagnosed injury [106]. Inability to explain the patient’s declining physiologic status should generally prompt an aggressive search for missed injuries, which may entail radiographic, angiographic and sonographic evaluation, and in some cases operative re-exploration.

After having ruled out occult injury and possible sources of ongoing hemorrhage, further lack of response to continued resuscitation may suggest exhaustion of physiologic reserves consistent with irreversible shock. Acidosis and hypothermia refractory to aggressive supportive measures, decreasing responsiveness to fluids or to vasopressors (“vasoplegia”), evidence of persistent hyperfibrinolysis on viscoelastic monitoring and diminished tissue oxygen saturation levels have all been suggested to correlate with likelihood of irreversible shock and non-survivable injury. While early prediction of mortality and organ dysfunction is possible, irreversible shock can generally only be identified after repeated and persistent efforts to resuscitate have proven unsuccessful. Futility may become an issue for these patients. The nature and severity of the injuries and the amount of resources already expended should certainly factor into the equation of when to discontinue further resuscitative efforts.

### Post-resuscitation complications in the trauma patient

As previously discussed, aggressive resuscitation of the polytrauma patient is not without the potential for significant complications. This section will focus systematically on commonly encountered clinical problems in the ICU that arise as a consequence of severe hemorrhagic shock and resuscitation, including complications of transfusion and fluid therapy.

#### Hypothermia

The development of hypothermia in trauma patients is complex and related to multiple factors including presence of shock, vasodilation from anesthetic agents, environmental exposure, infusion of large volumes of fluids, and surgical exposure [107,108]. Polytrauma patients presenting with uncontrolled, nontherapeutic hypothermia (<35°C) appear to have an associated increase in mortality [107,109-112] although this is an inconsistent finding in published studies [113]. Whether applied therapeutically or associated with severely injured trauma patients, hypothermia has multifactorial effects on the coagulation system with moderate hypothermia (32°C–34°C) reducing coagulation activity by 10% for every decrease in temperature by one degree Celsius as well as reducing the number and function of platelets [35,44,114]. In the setting of mild to moderate, controlled hypothermia (> 33°C), this degree of coagulopathy does not independently contribute to clinically significant bleeding [115]. During active hemorrhage and resuscitation, however, avoidance of severe hypothermia through active warming measures can be recommended based on the association of hypothermia with increased mortality as stated above.

In addition to alterations in coagulation, hypothermia has been associated with dysrhythmias and infections. Dysrhythmias may occur with moderate to severe hypothermia including bradycardia, first degree heart block and QT prolongation [116]. Although there are no consistent findings reported in the hypothermic trauma patient, continuous cardiac monitoring and evaluation of metabolic parameters is warranted. Infection risk for surgical site infections and pneumonia has been shown to be associated with hypothermia [117-119]. Although data to support a strong cause and effect relationship for hypothermia and increased morbidity and mortality, does not exist, our institution targets normothermia throughout the resuscitation and early ICU phase of care. Thus, active rewarming efforts initiated in the resuscitation bay or operating room are aggressively continued in the ICU. Targeted temperature management systems, preferably as part of an institutional protocol, can be used to achieve normothermia. Combination of different techniques including surface, intravascular, fluid, and forced-air warming systems provide a multi-modal approach to achieving and maintaining normothermia. A recent review of the relationship of hypothermia with acidosis and coagulopathy can be accessed for more detail [120].

#### Cardiopulmonary complications

##### TRALI

Transfusion-related acute lung injury (TRALI) is underreported and under-recognized, yet remains the leading cause of transfusion-related mortality [121,122]. TRALI is defined as an acute lung injury (ALI) that occurs during or within 6 hours of a transfusion, with no temporal relationship to alternative risk factors, and no evidence of circulatory overload [122-125]. As an ALI by definition, TRALI is associated with acute onset hypoxemia (PaO2/FiO_2_ gradient ≤ 300) and bilateral infiltrates on the chest radiograph [122,124]. “Possible TRALI” is the diagnostic nomenclature used when alternative explanations for ALI exist, such as aspiration pneumonitis, near drowning, lung contusion, or other trauma-related etiologies. TRALI is thought to be the result of two critical events: activation of the pulmonary vascular endothelium with priming of neutrophils, followed by transfusion of antibodies to leukocyte antigens with resultant activation and neutrophil-mediated cytotoxicity [126]. TRALI may occur as often as once for every 1271 units transfuse [127] with an incidence up to 8% in ICU patients [128]. Watson and colleagues estimated a cumulative increase in the risk of ALI from each unit of FFP at 2.5%, and of multiple organ failure from each unit at 2.1% [129].

Treatment for TRALI is supportive, and a lung-protective, low tidal volume strategy is recommended to prevent additional lung injury [130]. Subsequent transfusions should be limited when possible since repeated transfusions worsen outcomes in existing ALI [130,131]. If a restrictive transfusion strategy is not practicable, then avoidance of plasma from donors with pathogenic antibodies, administration of washed blood components, and use of products with the shortest length of storage possible are recommended [123]. In one study of 284 trauma patients, patients transfused with ABO-compatible plasma had over a 10% higher rate of acute respiratory distress syndrome [132]. Hence ABO identical blood products, as opposed to ABO-compatible products, should be used whenever possible.

##### TACO

Although the reported incidence varies widely, transfusion-associated circulatory overload (TACO) is currently the second most common cause of transfusion-related mortality [133,134]. TACO may be confused with TRALI since both conditions present with similar clinical and radiological findings. Large volume transfusions are not required for the development of TACO, which may particularly affect infants and the elderly. The key pathophysiologic difference between the two syndromes is lack of an antibody-mediated phenomenon with TACO [135]. Brain natriuretic peptide may be a helpful laboratory test for differentiating TACO from TRALI; levels are typically increased more than fourfold in the former [122]. Treatment consists of supportive care, diuretic therapy, and administration of any future transfusions at a reduced infusion rate.

Distinguishing TACO and TRALI from acute respiratory distress syndrome can be challenging since these disorders share several clinical characteristics, including the presence of bilateral, diffuse, infiltrates on the chest radiograph, and acute onset of respiratory distress and hypoxemia. Table 1 summarizes key criteria that may be used to differentiate these challenging disorders.

**Table 1:** Distinguishing TRALI from TACO and ARDS

**Finding**	**TRALI**	**ARDS**	**TACO**
Vital signs	May be febrile; hypotension more common than hypertension	Variable	Typically normothermic; hypertension
Clinical examination	Crackles	Crackles	Crackles, S3, jugular venous distension
ECHO findings	Normal to slightly decreased ventricular function; no evidence of left atrial hypertension	Normal to slightly decreased ventricular function; no evidence of left atrial hypertension	Decreased ejection fraction
Pulmonary artery occlusion pressure	< 18 mmHg	<18 mmHg	> 18mmHg
Fluid balance	Hyper-, hypo-, or normovolemic	Hyper-, hypo-, or normovolemic	Hypervolemic
Brain natriuretic peptide (BNP) level	< 200 pg/mL	< 200 pg/mL	> 1200 pg/mL
White blood cell count	Typically decreased; may be transient	Variable	Usually unchanged from baseline
Leukocyte antibodies	Donor leukocyte antibodies present; crossmatch incompatibility between donor and recipient	Donor leukocyte antibodies may or may not be present	Donor leukocyte antibodies may or may not be present
PaO_2_/FiO_2_ gradient	≤ 300	≤ 200	Variable

#### Renal and electrolyte complications

##### Rhabdomyolysis

Rhabdomyolysis is defined by the serum elevation of creatinine kinase (CK) as the result of destruction or disintegration of striated muscle [136]. Muscular trauma is the most common etiology, but the in some cases the cause can remain elusive. Heat stroke, inherited disorders of carbohydrate metabolism, electrical injuries, neuroleptic malignant syndrome, and medications can also cause rhabdomyolysis. Approximately 10–50% of patients with rhabdomyolysis develop acute renal failure [137,138]. CK and myoglobin levels are the most commonly used laboratory tests used to diagnose and monitor rhabdomyolysis. Normal CK levels are less than 260 U/L, and levels greater than 5000 U/L are associated with renal failure. With appropriate treatment, CK levels rise within 12 hours of injury, peak by 3 days, and fall 3–5 days afterwards [136]. Myoglobin has a half-life of less than 3 hours and may be a more sensitive laboratory indicator. Normal myoglobin levels are less than 1.5 mg/dL. Treatment of rhabdomyolysis consists of early and aggressive fluid therapy, with a target of 100 to 200 mL of urine per hour. Mannitol, bicarbonate, and various antioxidants are often used, but there are limited data to support the efficacy of these agents, and in these agents are generally avoided in the authors’ institution [136]. At least one study showed a benefit of forced diuresis with furosemide in casualties suffering from crush injuries [138]. Renal replacement therapy may be required, especially for patients with severe acidosis and hyperkalemia. It should be noted that during the renal recovery phase in rhabdomyolysis, hypercalcemia is a common electrolyte derangement; supplemental calcium should be avoided during this period unless hypocalcemia is symptomatic.

##### Hyperkalemia and hypocalcemia

While a comprehensive review of fluid and electrolyte management in the ICU is beyond the scope of this paper, a few electrolyte derangements unique to resuscitation from hemorrhage and severe shock are worth noting. Hyperkalemia can occur as the result of stored red blood cell membrane degradation, loss of cellular potassium pumps, and decreased adenosine triphosphate synthesis [139]. In one reported series, 16 patients who received red blood cell transfusions developed serum potassium levels between 5.9-9.2 mEq/L and sustained cardiac arrest [140]. Hyperkalemia should be treated promptly with insulin, glucose and calcium to protect the myocardium and increase intracellular potassium shifts. Emergent renal replacement therapy is indicated for life-threatening hyperkalemia, with or without concomitant renal failure. Hypocalcemia, owing to the binding of calcium to citrate preservatives in blood products, is another commonly encountered electrolyte derangement that may persist after admission to the ICU following damage control resuscitation. Hypocalcemia may impair hemostasis and contribute to hypotension, and should be promptly corrected if symptomatic.

#### Intra-abdominal hypertension and abdominal compartment syndrome

With the advances in DCS and DCR, an improved understanding of intra-abdominal hypertension (IAH) and abdominal compartment syndrome (ACS) has evolved [141]. IAH is defined as sustained or repeated pathologic elevation of intra-abdominal pressure ≥ 12 mmHg and ACS is defined as the sustained elevation of intra-abdominal pressures ≥ 20 mmHg that are associated with new organ dysfunction [142,143]. Risk factors for ACS include more than 3 L of crystalloid infusion or more than 3 units of PRBCs in the emergency department, hypothermia below 34°C, acidosis (base deficit < −14 mmol/L), and anemia (hemoglobin < 8 g/dL [144]. Additional factors include circulatory shock conditions and the amount of crystalloid fluids administered. Aggressive fluid resuscitation to targeted “supranormal” endpoints has been shown to result in an increased incidence of AC [91].

IAH and ACS have profound effects on multiple organ systems. Elevation of the intra-abdominal pressure to levels of 10 to 15 mmHg can cause cardiac failure as the result of inferior vena cava compression and compromised venous return [145]. Direct compression of the heart and pulmonary vessels results in elevated intra-thoracic pressures and a rightward shift and flattening of the Starling curve [145]. At intra-abdominal pressures as low as 10 mm Hg, oliguria may manifest as the result of compression of intrarenal blood vessels [143]. The low-pressure postglomerular intrarenal vascular network is highly sensitive to compressive forces; and renal artery blood flow has been shown in animal models to decrease in a linear fashion with increases in intra-abdominal pressure [146]. IAH and ACS can cause an imbalance between vasodilatory and vasoconstrictive mediators, mimicking the pathophysiology of hepatorenal syndrome, and causing hepatic insufficiency. Transmission of abdominal pressure to other compartments may result in the “multiple compartment syndrome” [147].

Successful management of IAH and ACS begins with prompt diagnosis. Physical examination has proven unreliable with a sensitivity of less than 60%, and the current standard of care is to measure intra-abdominal pressure by transducing urinary bladder pressure via an indwelling catheter [143,148]. Bladder pressures should be measured in the supine position at end-expiration, with the transducer zeroed at the iliac crest in the mid-axillary line [142,143]. Once diagnosed, there are few nonsurgical management options. Sedation, analgesia, and neuromuscular blockade, when combined with diuresis, fluid restriction, dialysis, or other interventions to attenuate hypervolemia, may avert the need to proceed to laparostomy. In many cases, prompt opening of the abdomen is the only effective intervention for restoring end-organ function [145,149]. Laparostomy practices have become more prevalent with the practice of DCS, with reported mortality improvements for severe abdominal trauma approaching 50% [3].

#### Hyperglycemia

Whereas hyperglycemia in ICU patients has remained a topic of debate over the past two decades, glucose elevations are very common in critically injured trauma patients, and tight glucose control has been associated with improved outcomes in this population [150]. In a retrospective cohort of 2,028 adult trauma patients, maintenance of blood glucose between 80–110 mg/Dl (4.4–5.6 mmol/L) utilizing an intensive insulin infusion protocol was associated with a decrease in hospital length of stay and mortality [151]. In another case control study, improved mortality was demonstrated in a trauma population when the blood glucose was maintained less than 150 mg/dL [152].

#### Adrenal insufficiency

Adrenal insufficiency has a high incidence amongst critically injured patients [153]. In one prospective observational study, up to 12% of all patients in shock had simultaneous hypothyroidism and adrenal insufficiency [154]. Trauma-related adrenal insufficiency has been correlated with systemic inflammatory response syndrome, and the multicenter HYPOLYTE study found that a continuous infusion of hydrocortisone (200 mg/d for 5 days, followed by a taper) resulted in a statistically significantly lower rate of hospital-acquired pneumonia (hazard ratio 0.51; 95% CI 0.30-0.83; p = 0.007) and hyponatremia [155]. The hydrocortisone group had more days of mechanical ventilation, but no differences regarding other infections, organ failure, or mortality were observed. The results of this study contrast with the CRASH study, which showed an increased risk of death in TBI patients treated with high-dose methylprednisolone [156]. Before the use of steroids can be widely recommended, the findings from HYPOLYTE should be confirmed with additional studies involving traumatically injured ICU patients, including TBI patients.

#### TRIM

Allogeneic blood transfusions introduce foreign antigens into the recipient, and can cause a constellation of beneficial or deleterious clinical effects that are collectively known as transfusion-related immunomodulation (TRIM) [123]. Though TRIM has been thought to have beneficial effects for renal allograft patients as evidenced by improved graft survival, deleterious effects in the trauma population include increased risk of infections and potentially higher mortality. The deleterious effects of TRIM on trauma patients are purportedly caused by soluble, white blood cell derived mediators accumulating in the supernatant fluid of stored red blood cells and soluble HLA peptides circulating in allogeneic plasma [123]. A host of pro-inflammatory effects are postulated in TRIM, leading to an increased incidence of postoperative bacterial infections, activation of endogenous cytomegalovirus or human immunodeficiency infection, and increased short-term mortality. TRIM appears to be a biological phenomenon that may be attenuated by the use of white blood cell (WBC) reduced blood products, although based on limited data from randomized controlled trials, universal WBC reduction is still not widely practiced.

Although CPDA red cell preservation techniques allow storage for up to 42 days, there is evidence that storage beyond 14 days significantly increases inflammatory mediators and by-products which may contribute to immune dysfunction [157,158]. Long-term storage also leads to red cell deformity which can cause trapping in the microcirculation and ischemia. Red cell hemolysis may lead to the accumulation of free radicals and to regional and systemic vasoconstriction, as well as oxidative injury [159]. These effects could potentially be synergistic with red blood cell damage and decreased micro-circulatory flow directly produced by hemorrhagic shock [160]. Duration of transfused red cell storage has been independently associated with an increased mortality and risk of multiple organ failure in trauma patients even with leukoreduction [159-163]. Although the data are mostly based on observational studies [159], trauma patients are the largest surgical consumers of blood products, and often receive the oldest blood. During acute resuscitation of massive hemorrhage it may not be feasible to choose only more recently stored units, but as transfusion requirements decrease the ICU physician may wish to be more selective.

### Summary

Polytrauma patients with severe shock from hemorrhage and massive tissue injury present major challenges for management and resuscitation in the intensive care setting. ICU physicians must be prepared to receive patients in varying degrees of stability and be ready to take over and complete the resuscitative process. In some cases “fine tuning” may be all that is required before addressing long-term critical care needs; in others, the intensivist must be prepared to undertake immediate massive resuscitation and correction of severe physiologic derangements if the patient is to survive the first 24 hours of admission. How well the ICU physician is prepared to meet these challenges may critically affect 24 hour survival after severe injury, and also the development of potentially life-threatening complications. As the patient becomes more fully stable, the ICU physician can then transition priorities towards longer-range management issues.

The past decade has seen radical changes in our approaches to resuscitation from hemorrhage and polytrauma. These changes have drastically influenced how resuscitation is carried out both in the resuscitation bay and operating room with carryover to the ICU. Future developments will improve our understanding of the micro-circulation and the interaction between hemostasis, inflammation and the endothelium and will influence how we manage these complex and challenging patients.

## Abbreviations

ACS: Abdominal compartment syndrome; ALI: Acute lung injury; CK: Creatinine kinase; CNS: Central nervous system; DCR: Damage control resuscitation; DCS: Damage control surgery; FFP: Fresh frozen plasma; IAH: Intra-abdominal hypertension; ICU: Intensive care unit; INR: International normalized ratio; PT: prothrombin time; PTT: Partial thromboplastin time; rFVIIa: Activated factor VIIa; TACO: Transfusion-associated circulatory overload; TBI: Traumatic brain injury; TRALI: Transfusion-related acute lung injury; TRIM: Transfusion-related immunomodulation.

## Competing interests

The authors declare that they have no competing interests.

## Authors’ contributions

All authors contributed to review concept, design and acquisition, analysis and interpretation of the literature. Finally all authors read and approved the submitted manuscript.
